# Supplementary material for: Closed-loop direct control of seizure focus in a rodent model of temporal lobe epilepsy via localized electric fields applied sequentially
Source: Nat Commun. 2022 Dec 17;13:7805. doi: 10.1038/s41467-022-35540-7 (PMC9759546; doi:10.1038/s41467-022-35540-7)
Supplement: Supplementary file 1 — Supplementary Information [file 41467_2022_35540_MOESM1_ESM.pdf]

Supplementary information

Title: Closed-loop direct control of seizure focus in a rodent model of temporal lobe epilepsy via localized electric fields applied sequentially

Wonok Kang[1,2], Chanyang Ju[2,3], Jaesoon Joo[4], Jiho Lee[2,3], Young-Min Shon[4,5,**], Sung-Min Park[1,2,3,6,7,*]

[1] School of Interdisciplinary Bioscience and Bioengineering, Pohang University of Science and Technology, Pohang, 37673, Republic of Korea

[2] Medical Device Innovation Center, Pohang University of Science and Technology, Pohang, 37673, Republic of Korea

[3] Department of Convergence IT Engineering, Pohang University of Science and Technology, Pohang, 37673, Republic of Korea

[4] Biomedical Engineering Research Center, Samsung Medical Center, School of Medicine, Sungkyunkwan University, Seoul, 06351, South Korea

[5] Department of Neurology, Samsung Medical Center, School of Medicine, Sungkyunkwan University, Seoul, 06351, Republic of Korea

[6] Department of Electrical Engineering, Pohang University of Science and Technology, Pohang, 37673, Republic of Korea

[7] Department of Mechanical Engineering, Pohang University of Science and Technology, Pohang, 37673, Republic of Korea

*** Corresponding author.**

Sung-Min Park, Ph.D.

Department of Convergence IT Engineering, Pohang University of Science and Technology, Pohang, 37673, Republic of Korea

Tel: +82-54-279-8842, Fax: +82-54-279-8859 E-mail: sungminpark@postech.ac.kr

**** Corresponding author.**

Young-Min Shon, MD, Ph.D.

Department of Neurology, Samsung Medical Center, School of Medicine, Sungkyunkwan University, Seoul, 06351, Republic of Korea

Tel: +82-2-3410-2701, Fax: +82-2-3410-0052 E-mail: youngmin.shon@samsung.com

Supplementary Figures

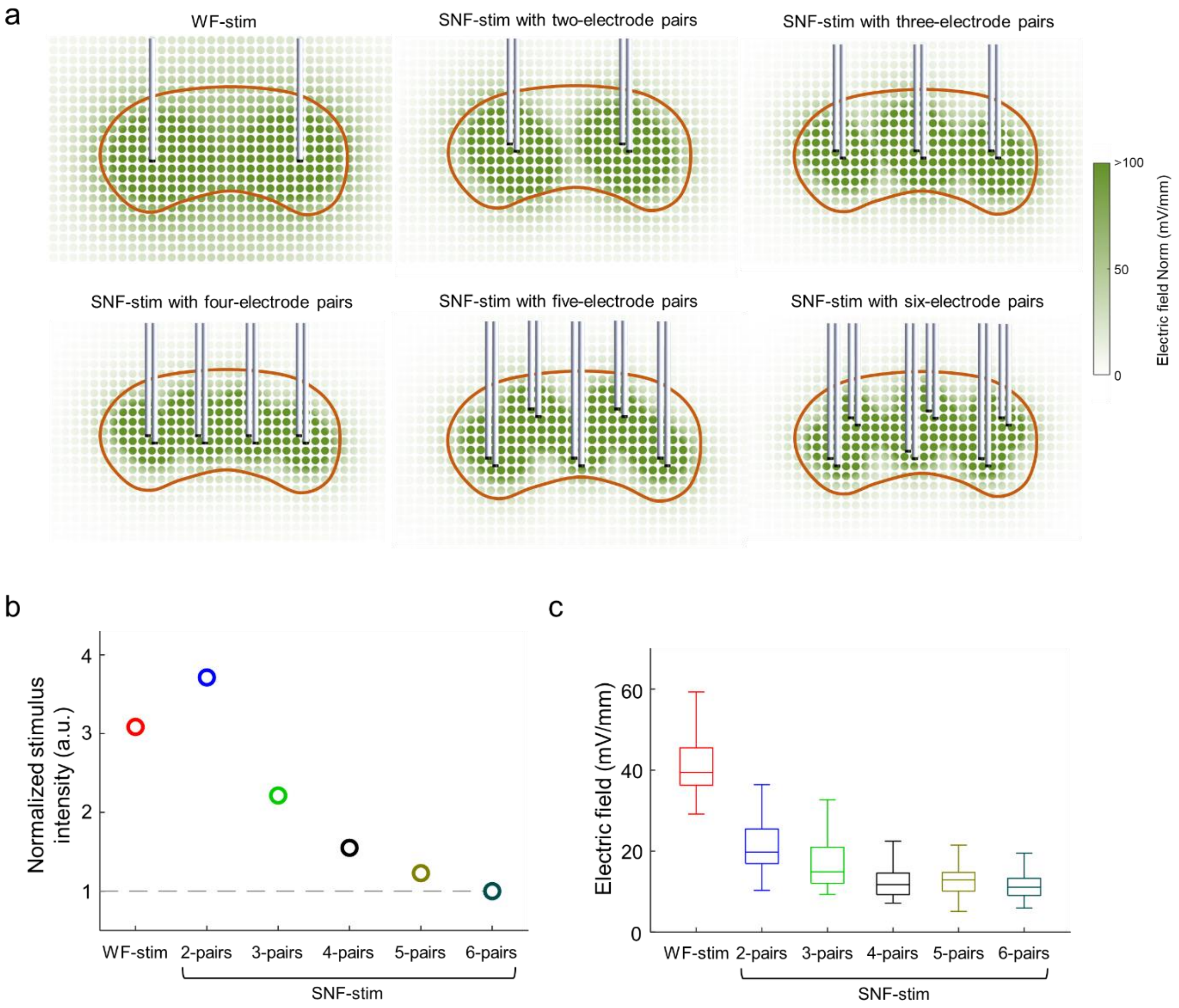

Supplementary Figure 1. Optimal electrode montage for SNF stimulation

a, Comparisons of induced electric fields during stimulation. The stimulus intensities were selected to achieve that electric fields of 100 mV/mm (or above) occupy 50% of the area of the target tissue while minimizing the off-target spreading fields. **b**, Injected stimulus current that was normalized to the intensity of the 6-pairs condition. **c**, Estimated spreading electric fields formed within 1mm outside the target, the hippocampus (n = 1660, simulated electric field intensity for each configuration). Center bar indicates median, box indicates 25th and 75th percentiles, and whiskers extend to the most extreme data points without outliers (±1.5 IQR). WF-stim, wide-field stimulation; SNF-stim, sequential narrow-field stimulation.

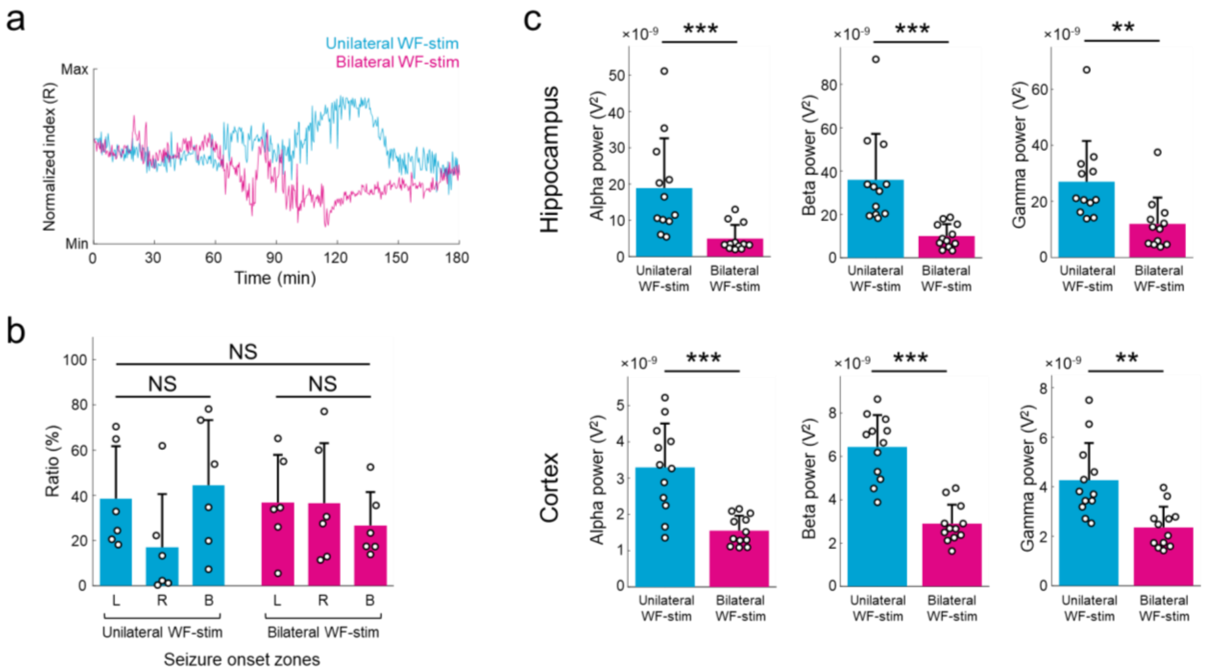

Supplementary Figure 2. Comparisons of seizure-suppressing effects during unilateral and bilateral WF stimulation

a, Representative result of calculated phase synchrony index during stimulation. **b**, Ratio of seizure onset sites upon stimulation methods, suggesting that there is no particular relationship between the onset zones and the type of stimulation ($P = 0.396$ for group comparisons, $n = 6$ rats per group, one-way ANOVA with Bonferroni correction). L, left hemisphere; R, right hemisphere; B, both hemispheres. **c**, Average band power for the alpha, beta, and gamma frequency from the hippocampus and cortex, respectively (P values in Supplementary Table 1, $n = 12$ in all comparisons, two-tailed Mann–Whitney *U* test). $^{**}P < 0.01$; $^{***}P < 0.001$; NS, not significant. Bar graphs depict data as mean ± SD. WF-stim, wide-field stimulation. For detailed statistical information, see Supplementary Table 1. Source data are provided as a Source Data file.

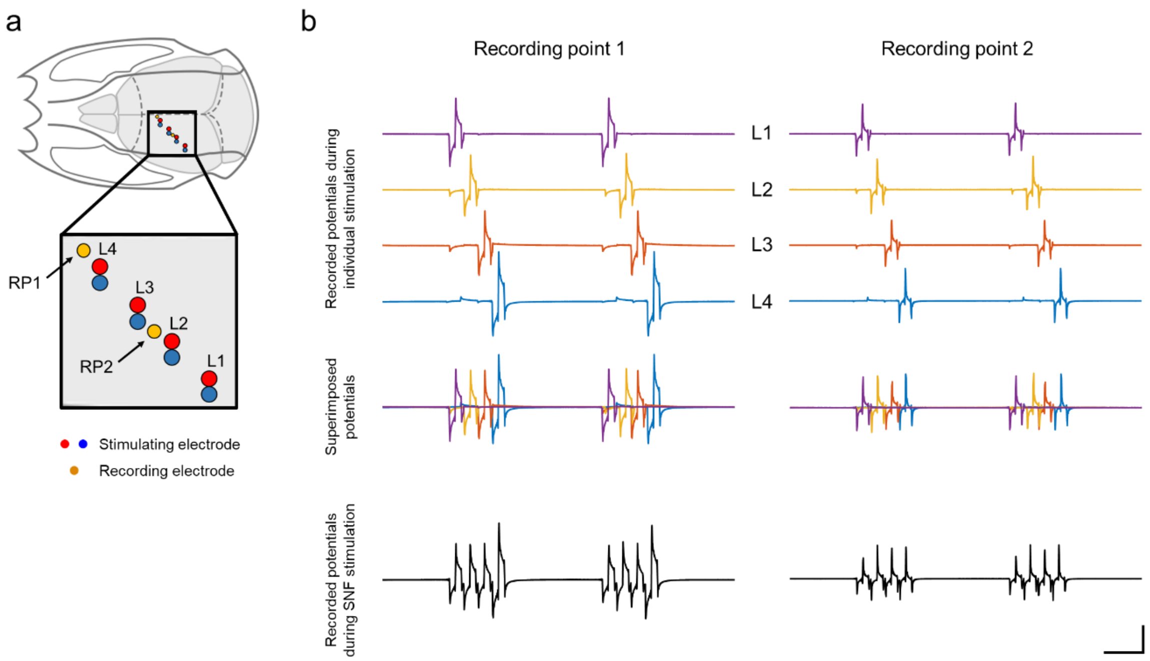

Supplementary Figure 3. Spatiotemporally superimposed electric potential during SNF stimulation

a, In vivo measurement setup to investigate electric potential changes over time during therapeutic intervention. L, stimulating electrodes implanted in the left hemisphere; RP, recording point of potential. **b**, Recorded potentials in RP1 and RP2 via individually applied stimulation (top). Simply superimposed potentials using potentials recorded during individual stimulation (middle). Induced potentials during SNF stimulation (bottom). Note that no effect or interference of sequentially induced gradients on each other compared with the superimposed data. Scale bar in **b**, 2 ms, 100 mV.

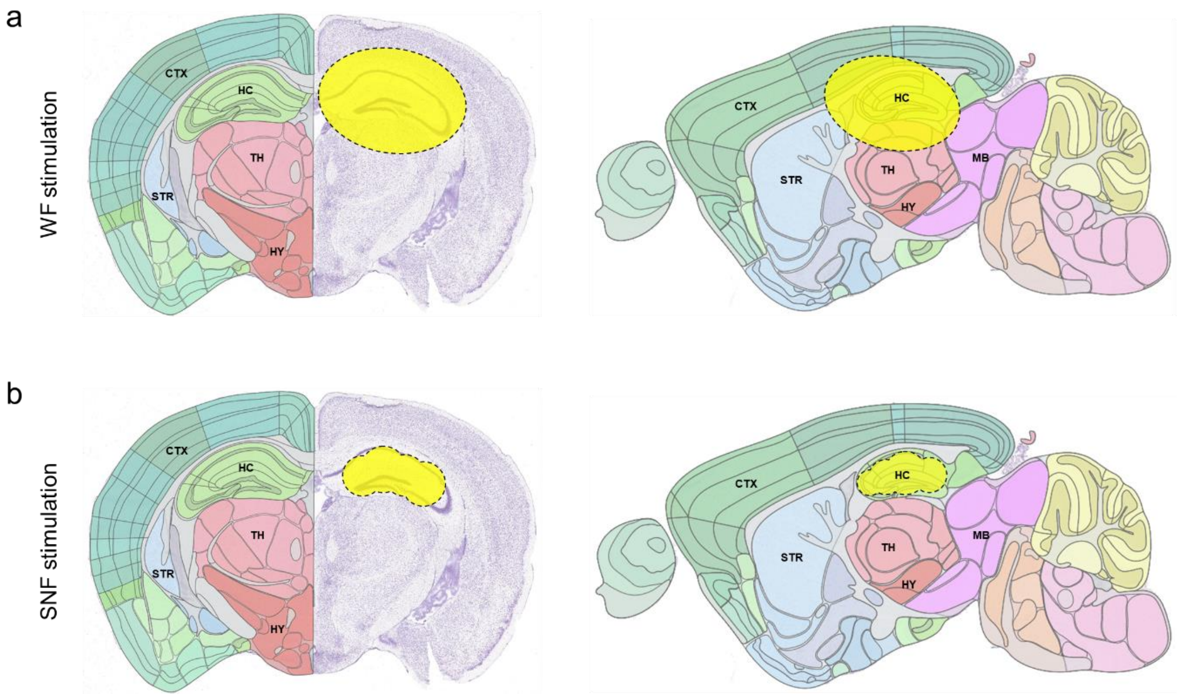

Supplementary Figure 4. Estimating activated area in hippocampal adjacent structures

Areas with electric field intensities of 100 mV/mm (or above) highlighted in yellow (**a**, **b**) both in coronal (left) and sagittal (right) views. The induced fields during WF stimulation (**a**) were significantly dispersed to the neighboring structures including the cortex, thalamus, midbrain, and striatum over SNF stimulation (**b**). Coronal and sagittal sections were adopted from Allen Mouse Brain Atlas© of Allen Institute for Brain Science available at https://mouse.brain-map.org/static/atlas[1].

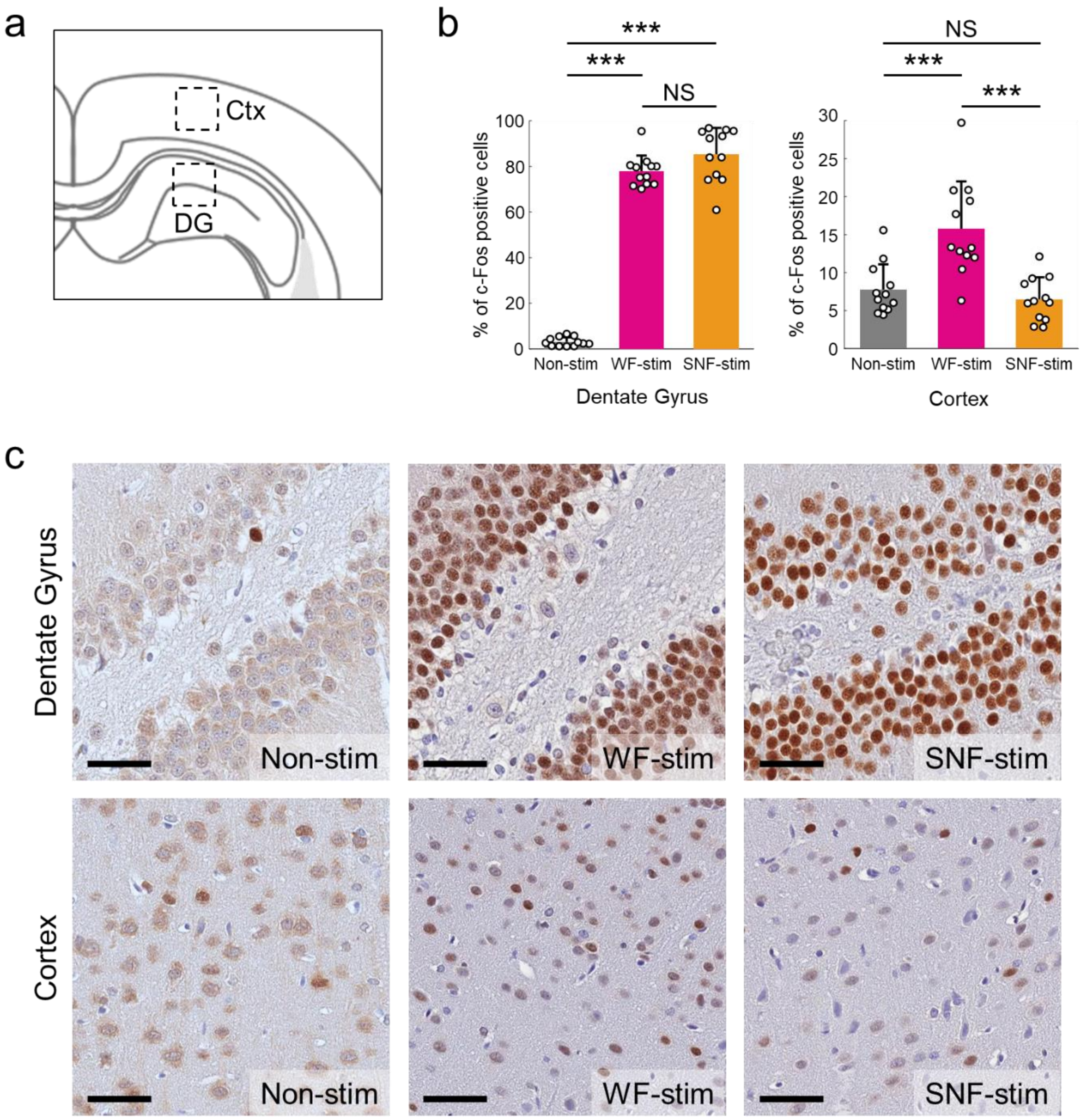

Supplementary Figure 5. Comparisons of c-Fos expression in normal rat brains

10 Hz WF and SNF stimulation (10 s on and 10 s off repetitively, for 20 min) was performed in normal rodent brains to determine the undesired neuronal activation due to the spreading fields (fixed 30 min after stimulation). **a**, Schematic depicting brain regions to be investigated. **b**, As expected, WF stimulation resulted in widespread c-Fos positive cells in both the cortex and hippocampus, whereas SNF stimulation only activated the cells in the dentate gryus, not the cortex compared to non-stimulated ones ($P < 0.001$ for all comparisons, $n = 12$ in all comparisons, one-way ANOVA with Bonferroni correction). **c**, Representative results of c-Fos expression in various conditions ($n = 4$ samples per group). ***$P < 0.001$; NS, not significant. Bar graphs depict data as mean ± SD. Scale bars in **c**, 50 μm. Non-stim, non-stimulation, WF-stim, wide-field stimulation; SNF-stim, sequential narrow-field stimulation. For detailed statistical information, see Supplementary Table 1. Source data are provided as a Source Data file.

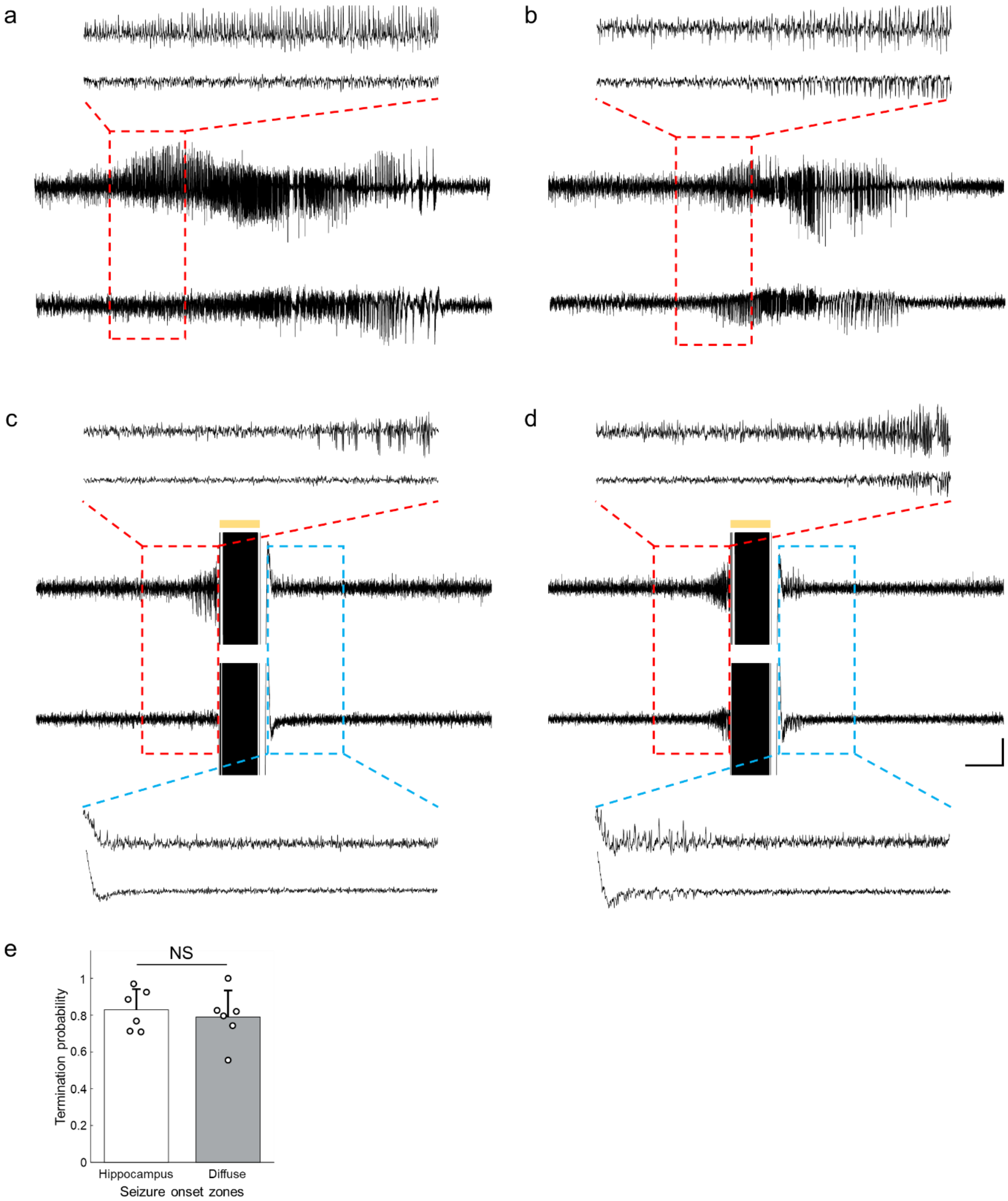

Supplementary Figure 6. Seizure suppression rates by seizure onset zones

Representative EEG traces with hippocampal onset (**a**) and diffuse onset (**b**) in the non-stimulation group. With SNF stimulation, epileptic rhythms were suppressed not only in hippocampal onset (**c**) but also in diffuse onset zones (**d**). **e**, Probability of terminating seizures for each seizure onset zone (0.83 and 0.79 for hippocampal and diffuse onset, respectively; P = 0.9362, n = 6 rats per group, two-tailed Mann–Whitney *U* test). NS, not significant. Bar graphs depict data as mean ± SD. Scale bars in **a**–**d**, 5 s, 100 µV. For detailed statistical information, see Supplementary Table 1. Source data are provided as a Source Data file.

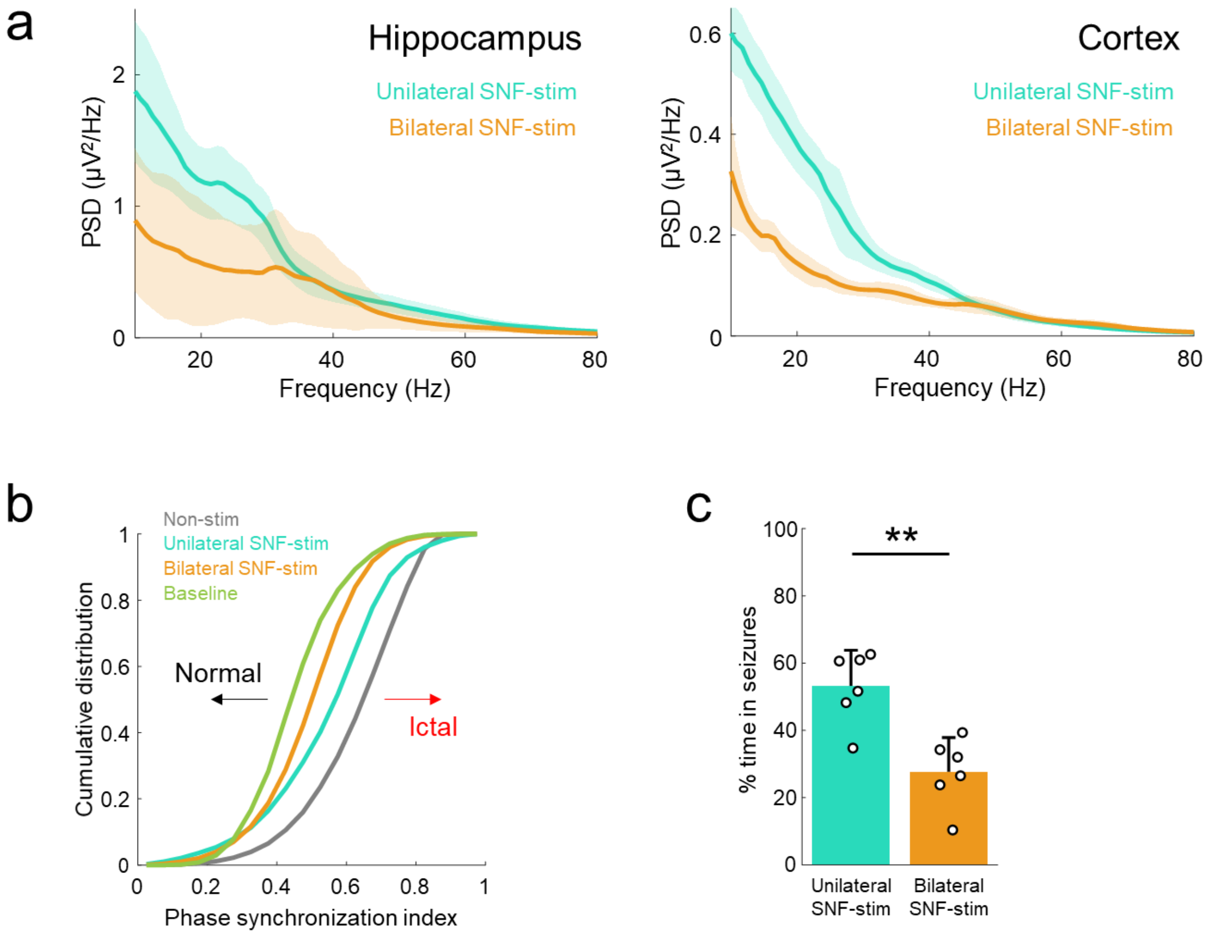

Supplementary Figure 7. Anti-seizure effects during unilateral versus bilateral SNF stimulation

a, Comparisons of PSD during unilateral and bilateral SNF stimulation. The bilateral SNF control significantly suppressed brain activities over the unilateral one. Lines represent the means and shaded areas represent SD. **b**, As expected, the bilateral SNF stimulation further desynchronized the ictal network over the unilateral condition. **c**, Total duration of seizure episodes during control (53.1% and 27.7% for unilateral and bilateral SNF conditions, respectively; P = 0.0082, n = 6 rats per group, two-tailed Mann–Whitney *U* test). **P < 0.01. Bar graphs depict data as mean ± SD. SNF-stim, sequential narrow-field stimulation. For detailed statistical information, see Supplementary Table 1. Source data are provided as a Source Data file.

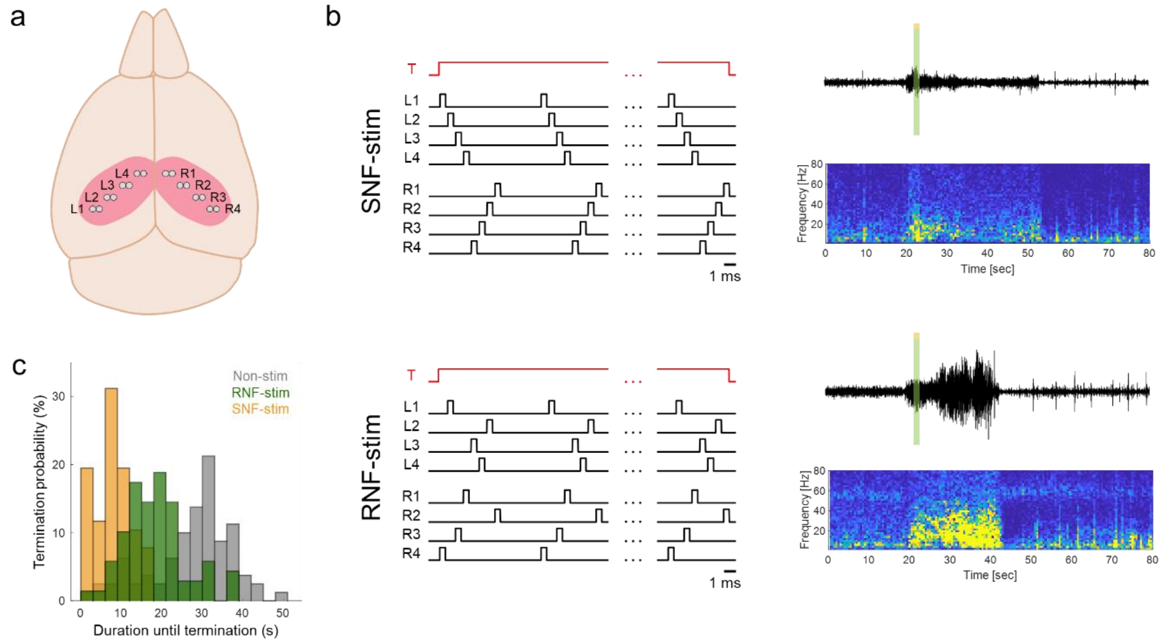

Supplementary Figure 8. Importance of sequential activation of target sites

a, Electrode configuration for in vivo animal test. **b**, Experimental protocol to compare the seizure-suppressing effects during SNF and RNF stimulation (left). The results showed that the spatiotemporally organized electric field has a superior seizure-inhibitory effect compared to the randomly applied micro-stimulation (right). **c**, Histograms of time taken to terminate seizures with treatment. Non-stim, non-stimulation; RNF-stim, random order narrow-field stimulation; SNF-stim, sequential narrow-field stimulation. Source data are provided as a Source Data file.

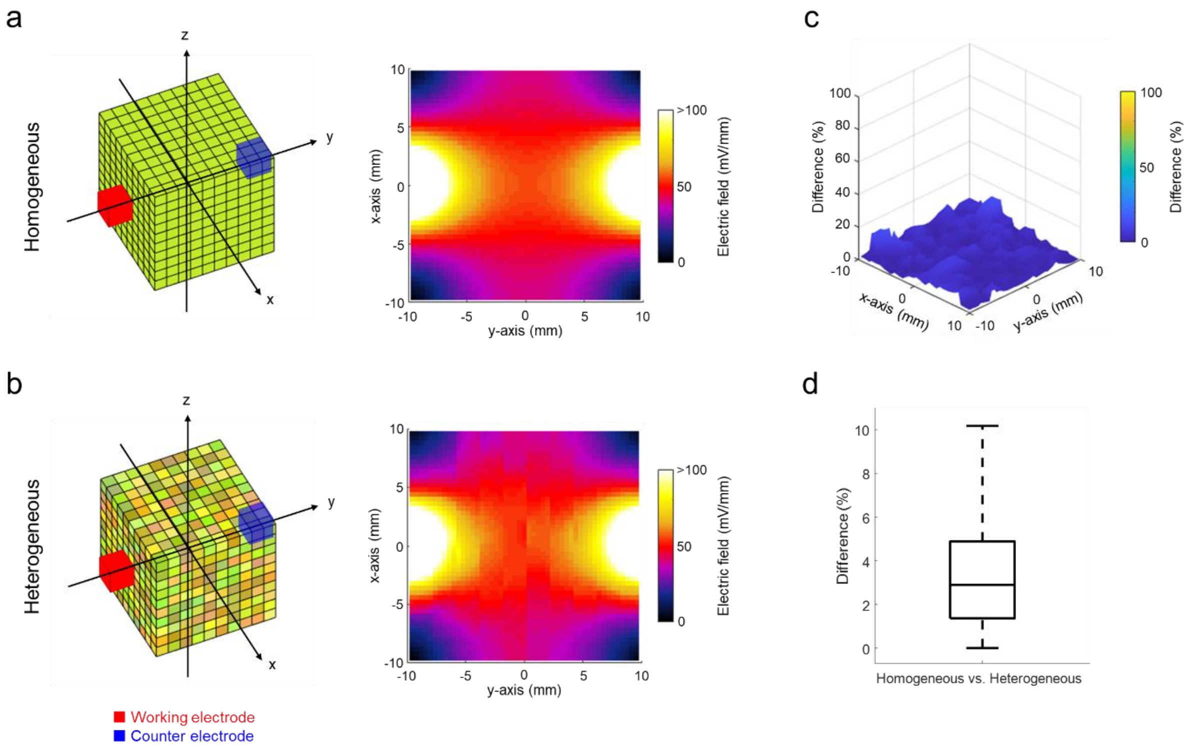

Supplementary Figure 9. Electric field estimation with homogeneous and heterogeneous electrical characteristics

a, Phantom with a single conductivity (0.7 S/m; left), and corresponding simulated field distribution in xy-plane (right). **b**, Phantom with randomly assigned conductivities (0.63–0.77 S/m; left), and predicted gradients (right). **c**, Example difference between results from homogeneous and heterogeneous conditions. **d**, Group results (n = 15600, difference in electric field distribution under one homogeneous versus six heterogeneous conditions, median: 2.90%, IQR = 1.36–4.89) Center bar indicates median, box indicates 25th and 75th percentiles, and whiskers extend to the most extreme data points without outliers (±1.5 IQR).

References

1. Lein, E. S. et al. Genome-wide atlas of gene expression in the adult mouse brain. Nature 445, 168–176 (2007).

Supplementary Tables

Supplementary Table 1. Statistical results

Figure	Panel	Statistical test used	Sample definition	Descriptive statistics shown	P value	Test statistic (D/F/t/z)
3	**f**	Two-way repeated measures ANOVA with Bonferroni correction	n = 12, immediate termination of seizures in 6 rats for each group	Full dataset & error bars are mean ±SD	P = 0.006 for two-way repeated measures ANOVA<br><br>Post-hoc test results<br>P = 0.1426, <0.001, 0.2620, 0.0722, 0.0222, 0.0037, <0.001, 0.0018, <0.001 for 20, 40, 60, 80, 100, 120, 140, 160, 180 min each	F(1, 22) = 9.474
	**g**	Two-tailed Mann–Whitney *U* test	n = 12, 6, 6, total seizure duration in 6 rats for each group	Full dataset & error bars are mean ±SD	P = 0.0226 for group comparison<br>P = 0.9362, 0.9362 for in-group comparison in unilateral and bilateral WF-stim conditions, respectively	z = -2.2805 for group comparison<br>z = 0.0801, -0.0801 for in-group comparison in unilateral and bilateral WF-stim conditions, respectively
4	**c**	One-way ANOVA with Bonferroni correction	n = 180, normalized field intensities in 6 rats	Box plots with whiskers	P < 0.001 for one-way ANOVA<br><br>Post-hoc test results<br>P = <0.001, <0.001, <0.001, <0.001 for R1, R2, R3, R4 in SNF configuration all vs. WF configuration<br>P = 1.00, 1.00, 1.00 for R2, R3, R4 vs. R1 in SNF configuration<br>P = 1.00, 0.6632 for R3, R4 vs. R2 in SNF configuration<br>P = 0.4113 for R3 v.s R4 in SNF configuration	F(4, 895) = 113.42
	**d**	One-way ANOVA with Bonferroni correction	n = 6, normalized area in 6 rats	Box plots with whiskers	P < 0.001 for one-way ANOVA<br><br>Post-hoc test results<br>P = <0.001, <0.001, <0.001, <0.001 for R1, R2, R3, R4 in SNF configuration all vs. WF configuration<br>P = 1.00, 1.00, 0.1703 for R2, R3, R4 vs. R1 in SNF configuration	F(4, 25) = 42.83

					P = 1.00, 0.0142 for R3, R4 vs. R2 in SNF configuration<br>P = 0.0311 for R3 v.s R4 in SNF configuration	
	**e**	One-way ANOVA with Bonferroni correction	n = 6, normalized area in 6 rats	Box plots with whiskers	P < 0.001 for one-way ANOVA<br><br>Post-hoc test results<br>P = <0.001, <0.001, <0.001, <0.001 for R1, R2, R3, R4 in SNF configuration all vs. WF configuration<br>P = 1.00, 1.00, 1.00 for R2, R3, R4 vs. R1 in SNF configuration<br>P = 1.00, 1.00 for R3, R4 vs. R2 in SNF configuration<br>P = 1.00 for R3 v.s R4 in SNF configuration	F(4, 25) = 82.79
	**h**	Kolmogorov–Smirnov test	n = 36, normalized field intensities in 4 rats	Cumulative histogram	P = <0.001, <0.001, <0.001, <0.001 for R1, R2, R3, R4 in SNF configuration all vs. WF configuration<br>P = 0.0489, 0.0138, 0.0215 for R2, R3, R4 vs. R1 in SNF configuration<br>P = 0.7992, 0.8972 for R3, R4 vs. R2 in SNF configuration<br>P = 1.00 for R3 v.s R4 in SNF configuration	D = 0.6733, 0.8020, 0.8911, 0.8812 for R1, R2, R3, R4 in SNF configuration all vs. WF configuration<br>D = 0.1881, 0.2178, 0.2079 for R2, R3, R4 vs. R1 in SNF configuration<br>D = 0.0891, 0.0792 for R3, R4 vs. R2 in SNF configuration<br>D = 0.0297 for R3 v.s R4 in SNF configuration
	**k**	Two-tailed paired *t*-test	n = 18, simulated thresholds at 18 positions	Full dataset shown	P < 0.001	t(17) = -22.8068
	**l**	Two-tailed Mann–Whitney *U* test	n = 6, measured thresholds in 6 rats for each group	Full dataset & error bars are mean ±SD	P = 0.0048	z = 2.8171
**5**	**c** left	Kruskal–Wallis test with Bonferroni correction	n = 12, alpha power from hippocampus in 6 rats for each group	Full dataset & error bars are mean ±SD	P(alpha) = 0.019<br>P(beta) < 0.001<br>P(gamma) = 0.0016<br>for Kruskal-Wallis test<br><br>Post-hoc test results<br>P(alpha) = 0.5444, 0.0194, 0.0166<br>P(beta) = 0.0029, 0.0014, 0.0351<br>P(gamma) = 0.0351, 0.0017, 0.0304<br>for Non-stim vs. LIWF-stim, Non-stim vs. SNF-stim, LIWF-stim vs. SNF-stim	$\chi^2(2)$ = 7.92<br>$\chi^2(2)$ = 15.42<br>$\chi^2(2)$ = 12.85<br>for alpha, beta, gamma power each<br><br>Post-hoc test results<br>z(alpha) = 0.6062, 2.3383, 2.3960<br>z(beta) = 2.9734, 3.2043, 2.1073<br>z(gamma) = 2.1073, 3.1466, 2.1651<br>for Non-stim vs. LIWF-stim, Non-stim vs. SNF-stim, LIWF-stim vs. SNF-stim
	**c** middle		n = 12, beta power from hippocampus in 6 rats for each group			
	**c** right		n = 12, gamma power from hippocampus in 6 rats for each group			

	**e** left	Kruskal–Wallis test with Bonferroni correction	n = 12, alpha power from cortex in 6 rats for each group		P(alpha) = 0.01<br>P(beta) < 0.001<br>P(gamma) < 0.001<br>for Kruskal-Wallis test<br><br>Post-hoc test results<br>P(alpha) = 0.0194, 0.0051, 0.8399<br>P(beta) = <0.001, <0.001, 0.1572<br>P(gamma) = 0.0011, <0.001, <0.001<br>for Non-stim vs. LIWF-stim, Non-stim vs. SNF-stim, LIWF-stim vs. SNF-stim	$\chi^2(2) = 9.22$<br>$\chi^2(2) = 24.29$<br>$\chi^2(2) = 26.63$<br>for alpha, beta, gamma power each<br><br>Post-hoc test results<br>z(alpha) = 2.3383, 2.8001, 0.2021<br>z(beta) = 4.1281, 4.1281, 1.4145<br>z(gamma) = 3.2620, 4.1281, 3.7239<br>for Non-stim vs. LIWF-stim, Non-stim vs. SNF-stim, LIWF-stim vs. SNF-stim
	**e** middle		n = 12, beta power from cortex in 6 rats for each group			
	**e** right		n = 12, gamma power from cortex in 6 rats for each group			
	**f**	Two-tailed Mann–Whitney *U* test	n = 6, immediate termination of seizures in 6 rats for each group	Full dataset & error bars are mean ±SD	P = 0.0202	z = 2.3219
	**g**	Kruskal–Wallis test with Bonferroni correction	n = 6, total seizure duration in 6 rats for each group		P < 0.001 for Kruskal-Wallis test<br><br>Post-hoc test results<br>P = 0.0051, 0.0051, 0.0051<br>for Non-stim vs. LIWF-stim, Non-stim vs. SNF-stim, LIWF-stim vs. SNF-stim	$\chi^2(2) = 15.16$<br><br>Post-hoc test results<br>z = 2.8022, 2.8022, 2.8022<br>for Non-stim vs. LIWF-stim, Non-stim vs. SNF-stim, LIWF-stim vs. SNF-stim
	**h**	Kruskal–Wallis test with Bonferroni correction	n = 6, seizure duration over segmented time in 6 rats for each group	Error bars are mean ±SD	P = 0.8665, 0.3123, 0.0117, 0.0018, 0.0007, 0.0006, 0.0005, 0.0005, 0.0014<br>for 20, 40, 60, 80, 100, 120, 140, 160, 180 min each<br><br>Post-hoc test results<br>P(Non-stim vs. LIWF-stim) = 0.8089, 0.2980, 0.0637, 0.0048, 0.0037, 0.0043, 0.0048, 0.0050, 0.0637<br>P(Non-stim vs. SNF-stim) = 0.8089, 0.2298, 0.0194, 0.0048, 0.0037, 0.0043, 0.0048, 0.0050, 0.0048<br>P(LIWF-stim vs. SNF-stim) = 0.6304, 0.4712, 0.0453, 0.1282, 0.0131, 0.0082, 0.0051, 0.0051, 0.0051<br>for 20, 40, 60, 80, 100, 120, 140, 160, 180 min each	$\chi^2(2)$ = 0.29, 2.33, 8.89, 12.59, 14.67, 14.9, 15.22, 15.17, 13.11<br>for 20, 40, 60, 80, 100, 120, 140, 160, 180 min each<br><br>Post-hoc test results<br>z(Non-stim vs. LIWF-stim) = 0.2419, 1.0408, 1.8545, 2.8220, 2.9057, 2.8526, 2.8220, 2.8072, 1.8545<br>z(Non-stim vs. SNF-stim) = 0.2419, 1.2010, 2.3383, 2.8220, 2.9057, 2.8526, 2.8220, 2.8072, 2.8220<br>z(LIWF-stim vs. SNF-stim) = -0.4812, 0.7206, 2.0016, 1.5212, 2.4820, 2.6421, 2.8022, 2.8022, 2.8022<br>for 20, 40, 60, 80, 100, 120, 140, 160, 180 min each
**6**	**b**	One-way ANOVA with Bonferroni correction	n = 10/10/12, 10/12/10, 10/12/12, 10/12/12 samples for CA1, CA3, DG, Ctx each, non-	Full dataset & error bars are mean ±SD	P = < 0.001, 0.0014, 0.32, <0.001<br>for CA1, CA3, DG, Ctx each	$F(2, 29) = 10.49$, $F(2, 29) = 8.29$,<br>$F(2, 31) = 0.32$, $F(2, 31) = 16.03$<br>for CA1, CA3, DG, Ctx each

			stim/LIWF-stim/SNF-stim in order, positive cells in brain sections from at least 6 rats for each group			
	**d**	One-way ANOVA with Bonferroni correction	n = 12/14/12, 12/14/12, 12/14/12, 12/14/12 samples for CA1, CA3, DG, Ctx each, non-stim/LIWF-stim/SNF-stim in order, positive cells in brain sections from at least 6 rats for each group	Full dataset & error bars are mean ±SD	P = 0.3426, 0.2648, 0.1138, 0.687 for CA1, CA3, DG, Ctx each	F(2, 35) = 1.1, F(2, 35) = 1.38, F(2, 35) = 2.31, F(2, 35) = 0.38 for CA1, CA3, DG, Ctx each
**7**	**b**	One-way ANOVA with Bonferroni correction	n = 12/14/12, 12/12/12, 12/14/12, and 12/14/12 samples for CA1, CA3, DG, Ctx each, non-stim/LIWF-stim/SNF-stim in order, positive cells in brain sections from at least 6 rats for each group	Full dataset & error bars are mean ±SD	P = 0.7833, 0.0392, 0.253, 0.7673 for CA1, CA3, DG, Ctx each	F(2, 35) = 0.25, F(2, 33) = 3.58, F(2, 35) = 1.43, F(2, 35) = 0.27 for CA1, CA3, DG, Ctx each
	**d**	One-way ANOVA with Bonferroni correction	n = 12/14/12, 12/12/12, 12/14/12, and 12/14/12 samples for CA1, CA3, DG, Ctx each, non-stim/LIWF-stim/SNF-stim in order, positive cells in brain sections from at least 6 rats for each group	Full dataset & error bars are mean ±SD	P = <0.001, <0.001, <0.001, <0.001 for CA1, CA3, DG, Ctx each	F(2, 35) = 28.66, F(2, 33) = 22.72, F(2, 35) = 19.29, F(2, 35) = 44.65 for CA1, CA3, DG, Ctx each
**8**	**c**	One-way ANOVA with Bonferroni correction	n = 80, 71, 78 for non-stim, LIWF-stim, SNF-stim each, seizure duration during non-stim, LIWF-stim, SNF-stim in 8 rats	Box plots with whiskers	P < 0.001 for one-way ANOVA<br><br>Post-hoc test results<br>P = <0.001, <0.001, <0.001<br>for Non-stim vs. LIWF-stim, Non-stim vs. SNF-stim, LIWF-stim vs. SNF-stim	F(2, 226) = 138.49
	**d**	Kolmogorov–Smirnov test		Cumulative histogram	P = <0.001, <0.001, <0.001<br>for Non-stim vs. LIWF-stim, Non-stim vs.	D = 0.2624, 0.2600, 0.2303<br>for Non-stim vs. LIWF-stim, Non-stim vs.

					SNF-stim, LIWF-stim vs. SNF-stim	SNF-stim, LIWF-stim vs. SNF-stim
	**f**	One-way ANOVA with Bonferroni correction	n = 5, time on stimulated area	Full dataset & error bars are mean ±SD	P < 0.001	F(5, 24) = 17
	**h**	One-way ANOVA with Bonferroni correction	n = 7, distance traveled in open field	Full dataset & error bars are mean ±SD	P < 0.001	F(5, 36) = 6.24
	**i**	One-way ANOVA with Bonferroni correction	n = 7, time in center in open field	Full dataset & error bars are mean ±SD	P < 0.001	F(5, 36) = 6.2
**Supp 2**	**b**	One-way ANOVA with Bonferroni correction	n = 6, seizure onset sites in 6 rats for each group	Error bars are mean ±SD	P = 0.1724 and 0.6546 for unilateral and bilateral WF stimulation in-group comparisons, respectively<br>P = 0.396 for group comparison	F(2, 15) = 1.98 and F(2, 15) = 0.44 for unilateral and bilateral WF stimulation in-group comparisons, respecitvely<br>F(5, 30) = 1.07 for group comparison
	**c** top	Two-tailed Mann–Whitney *U* test	n = 6, alpha, beta, and gamma power from hippocampus in 6 rats for each group	Error bars are mean ±SD	P = <0.001, <0.001, 0.0014<br>for alpha, beta, and gamma band each	z = 3.4352, 4.0703, 3.2043<br>for alpha, beta, and gamma band each
	**c** bottom	Two-tailed Mann–Whitney *U* test	n = 6, alpha, beta, and gamma power from cortex in 6 rats for each group	Error bars are mean ±SD	P = <0.001, <0.001, 0.0017<br>for alpha, beta, and gamma band each	z = 3.4930, 3.9548, 3.1466<br>for alpha, beta, and gamma band each
**Supp 5**	**b**	One-way ANOVA with Bonferroni correction	n = 12, c-Fos positive cells in brain sections	Full dataset & error bars are mean ±SD	P = <0.001, <0.001 for DG, Ctx each	F(2, 33) = 398.24, F(2, 33) = 15.53 for DG, Ctx each
**Supp 6**	**e**	Two-tailed Mann–Whitney *U* test	n = 6, seizure termination rates by seizure onset zones	Full dataset & error bars are mean ±SD	P = 0.9362	z = 0.0801
**Supp 7**	**c**	Two-tailed Mann–Whitney *U* test	n = 6, total seizure duration in 6 rats for each group	Full dataset & error bars are mean ±SD	P = 0.0082	z = -2.6421
