# Supplementary material for: Closed-loop direct control of seizure focus in a rodent model of temporal lobe epilepsy via localized electric fields applied sequentially
Source: Nat Commun. 2022 Dec 17;13:7805. doi: 10.1038/s41467-022-35540-7 (PMC9759546; doi:10.1038/s41467-022-35540-7)
Supplement: Supplementary file 3 — Reporting Summary [file 41467_2022_35540_MOESM3_ESM.pdf]

nature portfolio

Corresponding author(s): Young-Min Shon, Sung-Min Park

Last updated by author(s): Nov 29, 2022

Reporting Summary

Nature Portfolio wishes to improve the reproducibility of the work that we publish. This form provides structure for consistency and transparency in reporting. For further information on Nature Portfolio policies, see our Editorial Policies and the Editorial Policy Checklist.

Statistics

For all statistical analyses, confirm that the following items are present in the figure legend, table legend, main text, or Methods section.

n/a	Confirmed	
☐	☒	The exact sample size (n) for each experimental group/condition, given as a discrete number and unit of measurement
☐	☒	A statement on whether measurements were taken from distinct samples or whether the same sample was measured repeatedly
☐	☒	The statistical test(s) used AND whether they are one- or two-sided<br>*Only common tests should be described solely by name; describe more complex techniques in the Methods section.*
☒	☐	A description of all covariates tested
☐	☒	A description of any assumptions or corrections, such as tests of normality and adjustment for multiple comparisons
☐	☒	A full description of the statistical parameters including central tendency (e.g. means) or other basic estimates (e.g. regression coefficient) AND variation (e.g. standard deviation) or associated estimates of uncertainty (e.g. confidence intervals)
☐	☒	For null hypothesis testing, the test statistic (e.g. F, t, r) with confidence intervals, effect sizes, degrees of freedom and P value noted<br>*Give P values as exact values whenever suitable.*
☒	☐	For Bayesian analysis, information on the choice of priors and Markov chain Monte Carlo settings
☒	☐	For hierarchical and complex designs, identification of the appropriate level for tests and full reporting of outcomes
☒	☐	Estimates of effect sizes (e.g. Cohen's d, Pearson's r), indicating how they were calculated

Our web collection on statistics for biologists contains articles on many of the points above.

Software and code

Policy information about availability of computer code

Data collection	LabVIEW 2017 SP1 (EEG recording/trigger; real-time processing), Aperio ImageScope software v12 (histological image acquisition), Sim4Life v6.0 (3D simulation)
Data analysis	MATLAB R2019b, ImageJ 1.53j

For manuscripts utilizing custom algorithms or software that are central to the research but not yet described in published literature, software must be made available to editors and reviewers. We strongly encourage code deposition in a community repository (e.g. GitHub). See the Nature Portfolio guidelines for submitting code & software for further information.

Data

Policy information about availability of data

All manuscripts must include a data availability statement. This statement should provide the following information, where applicable:

- Accession codes, unique identifiers, or web links for publicly available datasets
- A description of any restrictions on data availability
- For clinical datasets or third party data, please ensure that the statement adheres to our policy

The data supporting the findings in this study are available within the paper and its Supplementary information/Source Data file. The raw data are available for research purposes from the corresponding authors on request. Source data are provided with this paper.

Human research participants

Policy information about [studies involving human research participants and Sex and Gender in Research.](#)

Reporting on sex and gender	Not applicable
Population characteristics	Not applicable
Recruitment	Not applicable
Ethics oversight	Not applicable

Note that full information on the approval of the study protocol must also be provided in the manuscript.

Field-specific reporting

Please select the one below that is the best fit for your research. If you are not sure, read the appropriate sections before making your selection.

☒ Life sciences ☐ Behavioural & social sciences ☐ Ecological, evolutionary & environmental sciences

For a reference copy of the document with all sections, see nature.com/documents/nr-reporting-summary-flat.pdf

Life sciences study design

All studies must disclose on these points even when the disclosure is negative.

Sample size	No statistical methods were performed to determined sample size a priori. Sample size was determined based on pilot experiments and other published studies addressing treatment of epileptic seizures (below references). Sample sizes for each experiment are reported in the main text and supplementary information.<br>Ref #1, Krook-Magnuson, E., et al. "On-demand optogenetic control of spontaneous seizures in temporal lobe epilepsy." Nature Communications 4, 1376. (2013)<br>Ref #2, Chen, Sin-Guang, et al. "Transcranial focused ultrasound pulsation suppresses pentylenetetrazol induced epilepsy in vivo." Brain Stimulation 13, 1. (2020)<br>Ref #3, Chen, R., et al. "Deep brain optogenetics without intracranial surgery." Nature Biotechnology 39, 161-164. (2021)
Data exclusions	We included all data from experiments except for stained results of brain tissue with broken anatomical landmarks.
Replication	Each experiment presented in this study was repeated independently in at least 4 animals (exact numbers as indicated in the main text and supplementary information).
Randomization	Rats were randomly assigned to control and experimental groups.
Blinding	Histological image acquisition was performed by an outsourcer, blind to experimental conditions. All data analyses were performed automatically using LabVIEW, MATLAB, and ImageJ, with the same code run on each experiment.

Reporting for specific materials, systems and methods

We require information from authors about some types of materials, experimental systems and methods used in many studies. Here, indicate whether each material, system or method listed is relevant to your study. If you are not sure if a list item applies to your research, read the appropriate section before selecting a response.

Materials & experimental systems

n/a	Involved in the study	
☐	☒	Antibodies
☒	☐	Eukaryotic cell lines
☒	☐	Palaeontology and archaeology
☐	☒	Animals and other organisms
☒	☐	Clinical data
☒	☐	Dual use research of concern

Methods

n/a	Involved in the study	
☒	☐	ChIP-seq
☒	☐	Flow cytometry
☒	☐	MRI-based neuroimaging

Antibodies

Antibodies used	Primary antibodies:<br>rabbit anti-c-Fos (ab209794, dilution 1:200, Abcam)<br>mouse anti-GAD65 (ab26113, dilution 1:100, Abcam)<br>rabbit anti-Iba1 (ab178846, dilution 1:2000, Abcam)<br>Secondary antibodies:<br>anti-mouse (K4001, dilution 1:2000, Agilent DAKO)<br>anti-rabbit (K4003, dilution 1:2000, Agilent DAKO)
Validation	We used commercial antibodies.<br>rabbit anti-c-Fos (ab209794, Abcam): 18 citations, PMID: 28059117, PMID: 28025994, (https://www.abcam.com/c-fos-antibody-ab209794.html).<br>mouse anti-GAD65 (ab26113, Abcam): 55 citations, PMID: 31664163, PMID: 28924206, (https://www.abcam.com/gad65-antibody-gad-6-ab26113.html).<br>rabbit anti-Iba1 (ab178846, Abcam): 161 citations, PMID: 33846335, PMID: 32284604, (https://www.abcam.com/iba1-antibody-epr16588-ab178846.html).

Animals and other research organisms

Policy information about studies involving animals; ARRIVE guidelines recommended for reporting animal research, and Sex and Gender in Research

Laboratory animals	Sprague Dawley male rats (250–350 g, 8-weeks old, ORIENT) were used in this study.
Wild animals	No wild animals were used in this study.
Reporting on sex	Since estrogens have been known to affect cognitive function and neurological diseases, only male rats were used.<br>Ref #1, Ciriza, Iratxe, et al. "Selective estrogen receptor modulators protect hippocampal neurons from kainic acid excitotoxicity: differences with the effect of estradiol." Journal of Neurobiology 61(2), 209-221. (2004)<br>Ref #2, Sato, Satoru M., and Catherine S. Woolley. "Acute inhibition of neurosteroid estrogen synthesis suppresses status epilepticus in an animal model." Elife 5, e12917. (2016)
Field-collected samples	No field samples were used in this study.
Ethics oversight	All experimental investigations were approved by the Institutional Animal Care and Use Committee (IACUC) at Pohang University of Science and Technology (approval number: POSTECH-2020-0083) and Samsung Biomedical Research Institute (approval number: 20220509001).

Note that full information on the approval of the study protocol must also be provided in the manuscript.
